# A review of the application and contribution of discrete choice experiments to inform human resources policy interventions

**DOI:** 10.1186/1478-4491-7-62

**Published:** 2009-07-24

**Authors:** Mylene Lagarde, Duane Blaauw

**Affiliations:** 1 Health Economics and Financing Programme, London School of Hygiene and Tropical Medicine, London, UK; 2 Centre for Health Policy, University of the Witwatersrand, Johannesburg, South Africa

## Abstract

Although the factors influencing the shortage and maldistribution of health workers have been well-documented by cross-sectional surveys, there is less evidence on the relative determinants of health workers' job choices, or on the effects of policies designed to address these human resources problems. Recently, a few studies have adopted an innovative approach to studying the determinants of health workers' job preferences. In the absence of longitudinal datasets to analyse the decisions that health workers have actually made, authors have drawn on methods from marketing research and transport economics and used Discrete Choice Experiments to analyse stated preferences of health care providers for different job characteristics.

We carried out a literature review of studies using discrete choice experiments to investigate human resources issues related to health workers, both in developed and developing countries. Several economic and health systems bibliographic databases were used, and contacts were made with practitioners in the field to identify published and grey literature.

Ten studies were found that used discrete choice experiments to investigate the job preferences of health care providers. The use of discrete choice experiments techniques enabled researchers to determine the relative importance of different factors influencing health workers' choices. The studies showed that non-pecuniary incentives are significant determinants, sometimes more powerful than financial ones. The identified studies also emphasized the importance of investigating the preferences of different subgroups of health workers.

Discrete choice experiments are a valuable tool for informing decision-makers on how to design strategies to address human resources problems. As they are relatively quick and cheap survey instruments, discrete choice experiments present various advantages for informing policies in developing countries, where longitudinal labour market data are seldom available. Yet they are complex research instruments requiring expertise in a number of different areas. Therefore it is essential that researchers also understand the potential limitations of discrete choice experiment methods.

## Background

The importance of human resources for health systems is demonstrated by ecological evidence of a positive correlation between the population density of health care providers in a country and the coverage achieved for cost-effective health interventions such as immunization or skilled attendance at delivery [1,2]. Several recent initiatives and reports have focused on the critical role played by human resources (HR) for health in improving health system performance [3-5]. It is now widely acknowledged that adequate health care delivery depends on the performance of the health workforce, which is determined by the availability, competence, productivity and responsiveness of health workers [4].

The so-called current human resources crisis pertains to all four dimensions of performance, but the issue of availability is particularly severe [4]. In the 2006 *World health report *on human resources for health, WHO identified 57 countries, most of them in sub-Saharan Africa, where there is a critical shortage of health care providers, defined as a density of health care professionals (counting only doctors, nurses and midwives) below 2.5 per 1000 population [4].

Furthermore, in all countries the shortage of health professionals is more critical in rural areas [6]. The geographical maldistribution of health workers exacerbates existing inequalities of access to basic health care and, therefore, contributes to lower health outcomes for the rural poor. Although this issue is more acute for developing countries, developed countries face similar problems of staff shortages and unequal distribution across their territories [7-11].

Various policies have been implemented in developed and developing countries to tackle these problems. Strategies involving direct financial incentives of one sort or another have constituted the majority of interventions [12-15]. Other related incentives have included the benefit derived from various training opportunities [16-18] or receiving financial help in exchange for a commitment to work in rural areas [13,14,19]. To date, no rigorous evaluation of the impact of these financial interventions has been carried out in developing countries [20], while in developed countries several studies have shown mixed results [13-15]. In recognition of the limitations of financial reward systems, some countries have opted for non-financial strategies such as encouraging the recruitment of students who appear more likely to work in rural areas after graduation [19,21-25], or increasing the sensitization of health students to rural areas [23,25-31]. Improving working conditions in remote areas has also been an objective for a number of policy interventions by, for example, allowing more flexible working arrangements [32,33], or improving access to communication [16,18,34,35].

Despite numerous initiatives, evidence on the effectiveness of these non-financial interventions is also limited [20] and HR problems persist everywhere. To better address these issues and craft adequate policy interventions, there is a need for further investigation into the nature and determinants of health workers' job choices. Indeed, current policy initiatives in many countries are hampered by the dearth of objective empirical data on health worker flows and behaviours, the determinants of their choices and the implications of these dynamics in terms of policy [20].

Various research tools have been used to investigate the factors driving the labour choices of health workers. A simple methodology is the use of cross-sectional organizational survey tools to measure outcomes such as job satisfaction, organizational commitment or intention to leave, and to evaluate individual and job characteristics that are correlated with those outcomes [7,36,37]. These studies have identified the range of factors, such as personal work ethic, remuneration, working conditions and career opportunities that influence the job choices of health workers but provide weak evidence on the relative importance of these different factors.

The availability of large health personnel datasets in certain developed countries has enabled the development of a second approach, based on econometric analyses of the determinants of the actual labour market decisions (revealed preferences) made by health workers during their careers [38-40]. This methodology does provide information on the relative importance of different individual and job characteristics that shape health workers' preferences and, therefore, is more useful in identifying potential human resource interventions. However, such research is not possible in most developing countries because longitudinal data on health personnel are either not available, or not detailed enough.

Lastly, a number of recent studies have used discrete choice experiments (DCE) to study the determinants of health workers' job preferences. Rather than evaluating the decisions that workers have actually made, this methodology analyses the stated preferences of health care providers for different job characteristics [41-44]. The investigation of stated preferences by means of discrete choice experiments has been used by researchers in other fields, such as marketing research and transport economics [45], to study the determinants of choices that cannot be easily observed in the market or for which a market does not exist [46].

Discrete choice experiments have become increasingly used in health services research, but primarily to assess patient-stated preferences and willingness to pay for different models of health care service delivery [47-50]. There are still only a small number of studies that have used this methodology to analyse the job preferences of health care providers. The aim of this article is to review the existing literature on the use of discrete choice experiments to study HR issues in both developed and developing countries. The intention is to draw lessons on the value of this relatively new methodology to inform HR policy development in developing countries. This paper first introduces the basic principles of DCE methods, then the methodology of our literature review is described. The main part of the paper describes the DCE studies we identified and summarizes their findings. The final discussion focuses on some cross-cutting lessons as well as the advantages and limitations of DCE methods for HR research.

## Methods

### Introduction to discrete choice experiments

There are a number of excellent reviews on the DCE methodology [45,51,52] and its application in health research [53-55]. Therefore, this section only provides a brief introduction to the basic principles for readers who are not familiar with this research method. Choice experiments are a quantitative methodology for evaluating the relative importance of the different product attributes that influence consumer choice behaviour [45]. This technique has its origins in the economic theory of demand, and especially in the work of Lancaster, who proposed that the demand for goods was effectively demand for their specific combination of product characteristics [56].

In choice experiments, respondents are asked to make choices between hypothetical alternative goods or services. Each good or service (or job description in HR applications) is described by several characteristics. Study objectives and preliminary work are usually key for the researcher to identify this limited set of characteristics that will be included [57,58]. In this paper we refer to these characteristics as attributes, while the combination of different attributes is termed a scenario. The responses given over a number of carefully selected scenarios enable the researcher to infer the relative importance of the different attributes.

According to random utility theory, individuals are assumed to choose the alternative that provides the highest individual benefit or utility. In the case of a binary choice between two different jobs, one would have:



where Y_k _is a choice variable that equals 1 when job *k *is chosen, and where U_ik_, the utility of individual *i *for job *k*, is assumed to be a function of the *n *attributes of the job:



Since evaluating all possible combinations of a set of product attributes would require an extremely large number of questions, a limited number is chosen, using experimental design techniques that ensure the selection of scenarios that optimize the information obtained from respondents. The selected scenarios are then organized into a series of choice sets. Each study participant then evaluates a number of choice sets and is asked to choose the preferred scenario (more details on DCE design can be found in other studies specifically addressing these methodological aspects – [55]).

DCE studies then use regression techniques to model respondents' choices as a function of the scenario attributes. Common modelling techniques used include random-effects logit or probit model and conditional logit models [53,54], while there is an increasing use of more sophisticated tools such as mixed logit models [59]. The significance and magnitude of the regression coefficients indicate the relative importance of those attributes that statistically influence respondents' choices. The negative of the ratio of any two coefficients represents the trade-offs made between the two attributes, and is called the marginal rate of substitution. When cost (or salary) coefficient is used as denominator, the ratio of coefficient provides an estimate of the willingness to pay for a particular characteristic. Performing regressions for different subgroups of health workers can be used to reveal differences in their preferences.

### Methods used for the review

Papers were primarily identified through a search of the following databases: Popline, PubMed, Econlit, HEED (Health Economics Evaluation Database), Emerald and Business Source Premier. These databases were used to cover most of the relevant literature from health and economics. Combinations of the following terms were used to search: "choice experiment", "discrete choice", "stated preferences", "human resources", "health personnel", "staff", "doctor", "nurse". A complementary search was made using Google Scholar.

In addition to the database searches, a snowballing approach was used to identify potential articles from the reference lists of relevant articles already identified. Some experts in the field of DCE and authors who had already published HR DCE studies were also contacted. The scope of the review included both developed and developing country research. However, only studies on health workers responsible for direct patient care were included in the review – so we focused on nurses, doctors and clinical officers but excluded a study on pharmacists [60] because we assumed that the nature of their work and their job preferences would be somewhat different.

The identified articles were compared in relation to study design, attributes included and key results. Although DCE results quantify the importance of attributes relative to one another, comparing the coefficients or marginal rates of substitution from one study to another is not valid (because an attribute's coefficient estimates are directly driven by all other included attributes). Therefore this overview presents a narrative synthesis and comparison of the findings of the identified studies.

## Results

### Description of studies

Nine studies were found that used DCE techniques to investigate HR problems. Four of them [44,61-63] were from the developed world (all in the United Kingdom) and the rest were carried out in developing countries: namely Indonesia [42], South Africa [41], Malawi [43], Ethiopia [64] and Tanzania [65]. The majority of study participants were doctors, while two studies focused on nurses, one reported results on both nurses and doctors [64] and one on clinical officers [65] (see Additional file 1).

Not all the studies clearly specified in their objectives that the design was meant to address issues related to health workforce maldistribution, but all did at least underline the direct relevance of the findings for policies tackling these issues.

In terms of DCE design, all studies employed an unlabelled experiment [51] with two choices: study participants had to choose between a generic job A and job B. They all used a fractional factorial (most of them creating 16 choice sets), and the predominant method to construct choice sets was to use a constant job scenario against which all other choice sets were compared.

The choice of attributes and levels was usually based on preliminary qualitative work and some literature review (Additional file 1). In most studies, pilot-testing the questionnaire enabled the researchers to refine the attribute levels and their wording. Despite the variety of attributes chosen, a few characteristics were common to all study contexts (Additional files 2 and 3). A salary variable was always present – not only because it is likely to be an important determinant of job preferences but also because it makes it possible to compute monetary equivalents for all other attributes in the DCE.

Beyond salaries, the range of attributes included in each study reflects elements identified in each context as determinants of health workers' motivation and choices. For example, in the United Kingdom, workload appeared as particularly important, and was included in different forms (hours worked per week, amount of after-hours work, patient list size, staffing levels). In developing countries, location appeared as a crucial job characteristic and was a DCE attribute in all but one study. Other recurring attributes were the provision of housing, the opportunity to benefit from further education, and improving the level of drugs and equipment in the facilities.

Finally, in all studies, the authors made use of one of the key strengths of DCE methods – the possibility of including job characteristics that are not currently present on the labour market, which could help identify policy levers to alter health workers' choices. In particular the range of salaries was often widened, which allowed to test the potential effects of an increase in remuneration. In some studies, certain job characteristics were completely different from existing possibilities in the labour market. For example in Indonesia, the authors delinked the opportunity to specialize from the obligation to do so in the public sector.

### Synthesis of findings

#### Studies from developed countries

Four studies reported findings for various groups of doctors in the United Kingdom. A study of general practitioners (GPs) in England [61] showed that they have the strongest preference for avoiding practices with higher levels of deprived patients. Larger list sizes were also disliked, while outside interests and working with an extended primary care team was favourably valued. Surprisingly, an increased out-of-hours work did not seem to diminish GPs' utility.

A study on GPs in the United Kingdom [44] provides evidence of the importance of non-financial job characteristics. More specifically, the author showed that several aspects of increased workload such as after-hours work, list size of patients and an increase in hours worked per week are disliked by GPs. In contrast, opportunities to develop interests outside work were valued positively by some subgroups of GPs.

Table 1, which reports some willingness-to-pay estimates of the job characteristics presented in these first two studies, highlights the similarities in some of their findings. For example, English GPs would want to be compensated by GBP 9 for each additional patient they took on their list, and GPs in the United Kingdom would have to be compensated by GBP 12 a year.

**Table 1:** Examples of monetary value of job characteristics

**Attribute**	**Gosden et al. 2000 **[61]	**Scott, 2001 **[44]
Opportunity to develop interest	-GBP 2269 to develop interest	+GBP 35 to develop interest

Out-of-hours worked (night shifts)	-GBP 402.67 for *some *hours done	+GBP 13 533 for some
		+GBP 19 708 for more

List size	+GBP 9 per additional patient	+GBP 12 per additional patient

Extended Primary Care Team	-GBP 2 393.30 for an extended team	

Administrative responsibilities	-GBP 1092 if no financial management responsibility	+GBP 1.10 per extra hour/year spent on administration

Change in *daytime *working hours	+GBP 701 per extra hour per week	+GBP 13 per extra hour per year

Use of guidelines		-GBP 3477 to use guidelines

Highly deprived patients	+GBP 5029 to work with such a population	
Moderately deprived patients	+GBP 1034 to work with such a population	

Note: A positive monetary value of a job characteristic can be interpreted as willingness to be compensated: it is the average salary increase needed to impose such a work characteristic. By contrast, a negative monetary value represents the salary cut respondents are ready to accept to benefit from the proposed job characteristic.

The strongest preference of consultants in Scotland [63] was to avoid being on-call after hours. They valued positively the opportunity to do non-NHS (National Health Service) work, having good working relationships with colleagues and higher staffing levels, but disliked longer weekly hours. The study also found that younger consultants were less likely to prefer on-call commitments, and that female consultants valued good relationships and the absence of staff shortages more.

Another study in Scotland [62] investigated differences in job preferences for two categories of doctors: sessional and principal GPs ("Principal GPs" refers to GPs who are full-time partners in a GP practice, whereas sessional GPs are employed by the practice and usually work part-time or are employed only for short periods, such as locums). In comparison to sessional GPs, the principal GPs valued continuing professional development and outside commitments more. Both groups were equally willing to avoid after-hours work and busier working weeks, and valued longer consultation times. Their study also emphasized that the gender and age of GPs reinforce some of these preferences (for example, women having a greater aversion for after-hours work).

#### Studies from developing countries

In developing countries, two studies reported findings on doctors, three on nurses and one on clinical officers.

Some results reported by Chomitz et al. [42] show significant differences in locational preferences across different groups of medical students in Indonesia. Male medical students from public schools and an urban background were not in favour of a position in a remote area, but valued the opportunity to specialize or to work in the public service. Male graduates from public schools and relatively rural backgrounds also valued remote locations negatively, but less so than their urban counterparts. However they did not attach a negative value to contract length. Male graduates from private schools and urban origins were strongly opposed to any time in the public service, while they favoured working in the province where they had studied. Female graduates from public medical schools and non-rural backgrounds showed higher disutility than men for positions offered in remote areas. Lastly, female graduates with rural origins attached a high value to being able to work in their birth region or the area where they studied.

However, the validity of these findings may be limited by the relatively small size of the subsamples used for each estimation and because the statistical significance of these results for each subgroup (compared to a model fitted for the whole population) was not presented in the report.

In a study in Ethiopia [64], Hanson and Jack show that the most important job characteristic for doctors was the possibility of working in the private sector (which was not allowed for public doctors at the time of the study). A pay increase was the next most-valued aspect of their jobs, followed by the provision of improved housing, being posted in Addis Ababa (compared to a regional city) or better equipment. Compulsory service in the public sector in exchange for training received was the least important preference. Subgroup analyses suggested that married doctors valued a job in Addis Ababa more, and that younger doctors were more impatient in wanting to be freed from their obligation towards the public sector after their training.

In the same study [64], Hanson and Jack reported the results of a similar choice experiment for nurses. Unlike doctors, the strongest preference for nurses was obtaining an increase in salaries, closely followed by the possibility of being posted in a regional capital. Also contrary to doctors' preferences was that nurses valued the availability of better equipment more than the opportunity of getting better housing for themselves, and the opportunity to work in the private sector came ahead of avoiding paying back years of training with years of work in the public sector. Interestingly, married nurses were less likely to prefer working in urban areas or the provision of housing than single nurses, which is somewhat unexpected and difficult to explain.

In another study on nurses in South Africa, Penn-Kekana et al. [41] found that earning twice as much money was the most attractive job characteristic. Better facility management and better equipment were next in importance. Being well staffed and having social amenities were the least important determinants of nurses' job choices. Subgroup analyses showed that younger nurses and those working in hospitals were more sensitive to salary levels, while nurses working in rural areas were more concerned about facility management.

A study on job choices of nurses working in the public sector in Malawi [43] shows that graduate nurses appreciated higher pay but also valued highly the opportunity to upgrade their qualifications quickly, as well as the provision of housing. Interestingly, nurses preferred jobs located in district towns compared to cities, and this preference was even stronger for nurses living in rural areas. Younger nurses also seemed less patient than older nurses in waiting for the opportunity to upgrade their qualifications.

Finally, the most recent of the identified studies investigated the preferences of clinical officers in Tanzania [65]. Salaries and education opportunities were found to be the most powerful incentives, but a better working environment (through improved infrastructure and equipment) was also valued. Interestingly, as in Malawi, the results indicated a willingness to avoid the capital city as a place of work, though district centres were still preferred to remote rural areas. This study also showed that people from rural backgrounds had less strong preferences than others for most job characteristics, and that women were less sensitive to pecuniary incentives and more concerned with facility upgrading than men were.

## Discussion

### Summary of findings

Certain methodological specificities limit the direct comparison and synthesis of the results obtained in the studies reviewed here. Indeed, study findings are dependent on the attributes included and influenced by the levels chosen in the experiment. In particular, some authors have argued that a distortion in the level range, for the salary variable in particular, could have important consequences for the results [66,67]. Furthermore, the comparison of results is also limited by variation in the choice of econometric models and differences in model specifications. For example, some studies have modelled the salary as a continuous variable, while others have treated different levels categorically though using dummy variables. This obviously modifies the interpretation of regression coefficients and the relative ranking of attributes. Despite these caveats, several findings emerge from this literature review.

Overall, the results from existing DCE studies on health workers' preferences show the relative importance of both pecuniary and non-pecuniary interventions (see Additional file 4). Non-financial strategies have the potential to make attractive incentives, and were often found to be more powerful than financial ones. This important finding is confirmed by a wide literature reviewing the effects of financial versus non-financial interventions [20,68].

DCE methods both build upon and complement other types of studies traditionally used in the HRH literature (see the brief overview in the introduction). On the one hand, DCEs rely partly on the findings from qualitative studies on job satisfaction to adequately define the range of attributes that will be relevant in the choice experiment. On the other hand, unlike studies based on ranking or rating methods, DCE studies force respondents to make trade-offs, thereby revealing and quantifying their underlying hierarchy of preferences. Although increased salaries always come up as a key determinant of job satisfaction, studies based on traditional questionnaires have failed to provide evidence of the relative importance of salary compared to other job characteristics. Using marginal rates of substitution, it is possible to compare the relative valuation of job characteristics in a DCE, as showed by the two examples reported in Table 1.

Finally, it should be noted that the studies reviewed here might be compromised by some methodological limitations. Some have criticized the lack of rigour in the experimental designs used by studies in the health economics literature [69]. Based on the information available in the articles, the studies summarized here are likely to have suffered from some flaws due to inappropriately constructed designs. For example, the use of a constant comparator in all but one study [65] suggests that they are unlikely to have used optimal designs [52].

### Implications for policy

Because DCE studies quantify the relative importance of determinants they provide more policy relevant information. All the studies reviewed here identified potential policy implications of their findings in each country context. Several aspects relating to quality of life (fewer hours per week and less after-hours work in developed countries; the provision of better housing and improved work conditions in developing countries) are positively valued by health workers in most countries, and can be therefore used as policy levers. Intellectual satisfaction in the United Kingdom, and education opportunities in developing countries also appear as important job characteristics that would increase the satisfaction of health workers.

In the more recent studies, most authors provided a list of possible interventions to influence health workers' job choices, particularly in addressing staff maldistribution and encouraging them to take up positions in under-served areas. By means of basic modelling techniques, the authors also computed the expected effects of such policies. For example, the study in Ethiopia [64] shows that the provision of a superior housing would increase the proportion of doctors willing to take up positions in rural areas by more than 250% (from 7.5% to 26.9%), while a reduction in time commitment to the public service would have a non-significant effect. Combined with an assessment of the costs of each package, such scenarios can provide essential estimation tools to approximate the relative cost-effectiveness of different HR incentives.

The results reviewed here also emphasize the importance of better understanding the preferences of different subgroups of health workers. This aspect is particularly crucial for health authorities to plan human resource interventions. Indeed, understanding the various aspirations and possible behaviours of subgroups of health workers might enable policy-makers to craft better policies to recruit and deploy health professionals where they are needed. Some studies reviewed here provide insights into such issues. For example, the recent study on clinical officers in Tanzania [65] shows that women are less responsive than men to pecuniary incentives, and tend to be more concerned with other working conditions (infrastructure, sufficient equipment). By providing information on how to target specific groups, and what would be the differential effects of policies for different subpopulations, DCEs are better suited than traditional forecasting tools used by policy-makers to predict health personnel needs in various geographical or professional areas.

### Implications for research

All the DCE studies we identified used unlabelled study designs, which assume that people value attributes equally across all job situations. For example, the studies from developing countries imply that health workers value a housing opportunity in urban and rural areas in the same way, or that they value the opportunity to work in the private sector equally in rural or urban areas. These are strong assumptions that could be investigated by the use of labelled or alternative-specific designs, which have often been used in transport or environmental economics. Such studies could also allow more flexibility and realism in the definition of the scenarios proposed, and be even more policy-relevant [70]. For example, the relative importance of job preferences across sectors (private versus public) could be explored with such designs. This question could be particularly relevant for countries where internal migration from the public to the private sector is a critical issue.

Stated preference methods have been critiqued because they may not predict real behaviours and choices. There is a growing literature in other fields trying to evaluate the correspondence between stated and revealed preferences [45,51]. In the field of HRH, the format of choice experiments, in asking respondents to choose their preferred job from two or more job descriptions, closely resembles the real decisions faced by individuals in their everyday life. As a result, health care markets are one of the areas where the comparison between revealed and stated preferences could be more common in the future, providing a richer set of data where choices made and options not selected would inform individual decision processes better.

Another field of investigation concerns the external validity of DCEs, which raises fundamental questions when interpreting the results of some of these studies. A complex issue is the extent to which the context and the individual experience have an impact on an individual's responses. As most DCE questionnaires present only very brief descriptions of attributes, there is some variation in how the attributes and levels will be interpreted by respondents. For example, Scott [44] mentions that the use of guidelines was valued positively by GPs, although researchers had expected this to be seen as a restriction to their autonomy. Clearly, respondents interpreted the attribute differently from what the researchers intended.

Similarly, several of the studies reviewed here from developing countries used rather imprecise terminology for job location, such as "city" or "rural", but it is not clear which characteristics the respondents associated with those labels. Finally, when using job attributes currently not available on the job markets (such as potential policy interventions), it is difficult to assess the extent to which respondents will be able to easily appreciate or believe those possibilities. These are examples of areas where other research methods, such as qualitative tools, could be helpful in understanding the results of the DCEs better, and take the debate on the limitations and interpretation of DCEs forward.

## Conclusion

Although choice experiments have become an increasingly popular technique in the field of health economics, to date they have been less commonly used in developing country contexts, although there are studies in developing countries from disciplines other than health economics [71-73]. DCEs could be a particularly valuable method in the field of human resources research in developing countries, where reliable retrospective datasets are quite scarce and prospective studies are needed to support planning decisions. DCE is appealing because it seems to provide policy-relevant information and may constitute a cheap and quick method to investigate the relevance of potential policy options. This is particularly appealing in developing country contexts, where detailed evaluations of policy interventions or rich datasets on health worker career paths are rare [20] and would be costly to implement. Specifically in developing countries, these techniques could help policy-makers craft policies to reduce public-private and rural-urban maldistribution.

However, the application of this methodology is relatively complex, as the construction of choice experiments requires the understanding and application of advanced notions in experimental design theory [52]. Although some software programs, such as SAS [74], now provide tools to help researchers construct optimal designs, proper experimental design remains a very technical and evolving field that might limit the use of DCE methods.

A large literature devoted to choice experiments has already underlined some of the limitations of certain study designs [51], while design constraints or limitations can limit the validity of results obtained from such choice experiments [66,67,75,76]. The econometric analysis of DCE data also requires fairly advanced statistical methods and there is no consensus in the literature at present on the best models to use [53,77]. Although choice experiments may be useful in informing decision-making in developing countries, HR researchers should be aware of the technical expertise required to use them, as well as their potential limitations.

## Competing interests

The authors declare that they have no competing interests.

## Authors' contributions

ML participated in the design of the study, carried out some of the literature searches, synthesized the findings and drafted the manuscript. DB participated in the design of the study, carried out some of the literature searches, and edited the manuscript. Both authors read and approved the final manuscript.

## Supplementary Material

Additional file 1 **Study characteristics**. Microsoft Word table in landscape format. Click here for file

Additional file 2 **Attributes and levels of choice experiments implemented in developing countries**. Microsoft Word table in landscape format. Click here for file

Additional file 3 **Attributes and levels of choice experiments implemented in developed countries**. Microsoft Word table in landscape format. Click here for file

Additional file 4 **Ranking of attributes according to their importance**. Microsoft Word table in landscape format. Click here for file
