# Supplementary material for: A review of the application and contribution of discrete choice experiments to inform human resources policy interventions
Source: Hum Resour Health. 2009 Jul 24;7:62. doi: 10.1186/1478-4491-7-62 (PMC2724490; doi:10.1186/1478-4491-7-62)
Supplement: Additional file 1 — Study characteristics. Microsoft Word table in landscape format. [file 1478-4491-7-62-S1.doc]

### Additional file 1: Study characteristics

| **Authors and date** | **Ref.** | **Study Setting** | **Study participants** | **Objective of the study** | **Number of attributes** | **Number of choice sets in fractional factorial** | **Number of choices made by each respondent** | **Administration of questions** | **Choice of attributes was based on** |
| --- | --- | --- | --- | --- | --- | --- | --- | --- | --- |
| Chomitz et al. 1998 | [42] | Indonesia | 585 final-year medical students | To understand doctors’ preferences regarding various possible incentives, in particular to attract them to rural or remote places | 6 | 18 | 18 | Authors mention 50 sets of 18 pair-wise tasks 9 prohibited combinations | N/A |
| Gosden et al. 2000 | [61] | England | 172 GPs | To investigate GP preferences for practice and job characteristics, in order to understand what factors might improve GP recruitment in under-served areas | 8 | 27 | 6 or 7 | 1 constant comparator; 4 blocks of 6 or 7 pair-wise choices | Literature review, in-depth interviews and FGDs |
| Scott, 2001 | [44] | UK | 1206 GPs | To investigate GPs’ preferences for financial and non-financial incentives | 7 | 18 | 4 or 5 | 1 constant comparator; 4 blocks of 4 or 5 pair-wise choices | Literature review, in-depth interviews |
| Ubach et al. 2003 | [63] | Scotland | 2923 Consultants | To examine the strength of hospital consultants’ preferences for various aspects of their jobs to improve recruitment and retention | 6 | 16 | 6 | 1 constant comparator used; 3 blocks of 6 pair-wise choices (5 + 1 to test rationality) | Literature review, in-depth interviews and FGDs |
| Wordsworth, 2004 | [62] | Scotland | 904 principals & 388 sessional GPs | To identify the relative value given by sessional GPs to various job characteristics, in order to inform issues on recruitment and retention of GPs | 7 | 16 | 6 | 1 constant comparator used; 3 blocks of 6 pair-wise choices (5 + 1 to test rationality) | Literature review, in-depth interviews and FGDs |
| Penn-Kekana et al. 2004 | [41] | South Africa | 147 maternity nurses | To explore the relative importance of various job characteristics to explain staff dynamics | 5 | 15 | 15 | 1 constant comparator used across 15 (pair-wise) choice sets | FGDs |
| Mangham & Hanson, 2007 | [43] | Malawi | 107 registered nurses | To determine the range and relative importance of various factors that affect nurses’ job choices in the public sector | 6 | 16 | 15 | 1 constant comparator used across 15 (pair-wise) choice sets | Mostly FGDs and in-depth interviews |
| Hanson and Jack 2008 | [64] | Ethiopia | 216 doctors | To estimate the effects of possible policy interventions to improve the supply of doctors in rural areas | 6 | 16 | 15 | 1 constant comparator used across 15 (pair-wise) choice sets ; 4 question orders | Interviews with officials, and FGDs held in Malawi |
| Hanson and Jack 2008 | [64] | Ethiopia | 640 nurses | To estimate the effects of possible policy interventions to improve the supply of nurses in rural areas | 6 | 16 | 15 | 1 constant comparator used across (pair-wise) 15 choice sets | Interviews with officials, and FGDs held in Malawi |
| Kolstad 2008 | [65] | Tanzania | 320 clinical officers | To estimate clinical officers’ job preferences in order to understand how rural jobs can be made more attractive | 7 | 32 | 16 | 32 choice sets divided into 2 blocks of 16 pairs (4 sets of questionnaires with random order of questions) | Literature review, in-depth interviews |

Note: N/A indicates that the information is not available from the original article.
