# Supplementary material for: A review of the application and contribution of discrete choice experiments to inform human resources policy interventions
Source: Hum Resour Health. 2009 Jul 24;7:62. doi: 10.1186/1478-4491-7-62 (PMC2724490; doi:10.1186/1478-4491-7-62)
Supplement: Additional file 2 — Attributes and levels of choice experiments implemented in developing countries. Microsoft Word table in landscape format. [file 1478-4491-7-62-S2.doc]

### Additional file 2. Attributes and levels of choice experiments implemented in developing countries

| **Authors and date** | **Ref.** | **Job location** | **Remuneration** | **Contract duration** | **Staffing/ workload** | **Continuing Education/career development** | **Career Development** | **Outside work opportunities** | **Resource constraints** | **Management** | **Housing** | **Other living conditions** |
| --- | --- | --- | --- | --- | --- | --- | --- | --- | --- | --- | --- | --- |
| Chomitz et al, 1998 | [42] | Province  7 provinces  Remoteness  Non-remote  Remote  Very remote | Total monthly income  Rp 500,000  Rp 1,000,000  Rp 1,500,000  Rp 2,000,000  Rp 2,500,000  Rp 3,000,000 | Length of contract  1 yr  2 yrs  3yrs |  | Probability of subsequent specialist training  10 - 90% | Probability of subsequent civil service appointment  10 - 90% |  |  |  |  |  |
| Penn-Kekana et al, 2004 | [41] |  | Salary  Same  15% more  Double |  | Staffing  Under-staffed  Well-staffed |  |  |  | Equipment  Poorly equipped  Fully equipped | Facility management  Poor  Good |  | Social Amenities  Under-developed  Developed |
| Mangham & Hanson, 2007 | [43] | Place of work  City  District town | Net monthly pay  K 30,000  K 40,000  K 50,000 |  | Typical daily workload  Light  Medium  Heavy | Opportunity to upgrade qualifications  After 3 yrs  After 5 yrs |  |  | Material availability  Inadequate  Adequate |  | Provision of housing  None  Basic  Superior |  |
| Hanson and Jack 2008 | [64] | Place of work  City  Rural | Salary  “Average” salary  1,5 x base salary  2 x base salary | Commitment following training  1 year / yr training  2 years / yr training |  |  |  |  | Equipment & drug availability  Inadequate  Improved | Supervision  High  low | Provision of housing  None  Basic  Superior |  |
| Hanson and Jack 2008 | [64] | Place of work  Addis Ababa  Zonal capital | Salary  “Average” salary  1,5 x base salary  2 x base salary | Commitment following training  1 year / yr training  2 years / yr training |  |  |  | Permission to work in the Private Sector  Yes  No | Equipment & drug availability  Inadequate  Improved |  | Provision of housing  None  Basic  Superior |  |
| Kolstad 2008 | [65] | Location  Dar-es-Salaam, region HQ, district HQ,  at least 3 hrs from district HQ | Salary  TSH 650,000  TSH 500,000  TSH 350,000 |  | Workload  Normal (1h extra /day)  Heavy (3hrs extra /day) | Education opportunities  No education offered  Education offered after 2 years / 4 years / 6 years of services |  |  | Equipment & drug availability  Sufficient  Insufficient |  | Provision of housing  Decent house  No house provided | Infrastructure  Reliable mobile coverage, electricity and water  Unreliable mobile coverage, electricity and water |
