# Supplementary material for: A review of the application and contribution of discrete choice experiments to inform human resources policy interventions
Source: Hum Resour Health. 2009 Jul 24;7:62. doi: 10.1186/1478-4491-7-62 (PMC2724490; doi:10.1186/1478-4491-7-62)
Supplement: Additional file 3 — Attributes and levels of choice experiments implemented in developed countries. Microsoft Word table in landscape format. [file 1478-4491-7-62-S3.doc]

### Additional file 3. Attributes and levels of choice experiments implemented in developed countries

| **Authors and date** | **Ref.** | **Remuneration** | **Staffing** | **Workload** | **Nature of work** | **Administrative load** | **Overtime work** | **Continuing Education/ Career Development** | **Outside work opportunities** | **Independence** | **Workplace culture** |
| --- | --- | --- | --- | --- | --- | --- | --- | --- | --- | --- | --- |
| Gosden et al. 2000 | [61] | Change in income from current work  £ 7,500 more  £2,500 more  £2,500 less | Size of the primary health care team  Skeleton  Extended | List size per GP  1800 patients  2000 patients  2200 patients  Change in hours worked/week (from present post)  2,5 hours more  2,5 hours less  7,5 hours more | Level of deprivation among patients in practice population  Low  Medium  High | Who does the majority of financial management  You  A practice manage/other | Out of hours done  Some  None |  | Opportunities to develop outside interests  No  Yes |  |  |
| Scott, 2001 | [44] | Change in income per year  £ 2,500 less  No change  £ 2,500 more |  | List size per GP  1400 patients  2000 patients  2600 patients  Total daytime hrs worked per week  35  40  45 |  | Time spent on administration / week  7 hrs  10 hrs  13 hrs | Out of hours arrangements  No nights or weekends  1 night/wk+ 1wkend/6  2 nights/wk+1 wkend/4 |  | Opportunities to develop special interests or academic work  Yes  No | Use of guidelines  Yes  No |  |
| Ubach et al. 2003 | [63] | Salary  Same  +10% more  +20% | Staffing  Enough staff  Shortage of staff | Change in hours worked/week  10 Hrs less  5 Hrs less  No change  5 Hrs more |  |  | On Call duties  Home not busy  Home very busy  Residential not busy  Residential very busy |  | Opportunity to do non-NHS medical work  None  Some  Unlimited |  | Working relationships  Good  Fair |
| Wordsworth, 2004 | [62] | Salary  Same  +10% more  +20% |  | Change in total hrs worked / week  5 hrs less  No change  5 hrs more | Consultation length  5 mins  10 mins  15 mins | Involvement in practice decisions  None  Some  Extensive | Out-of-hours work  Low  Medium  High | Professional development & training  Enough  Not enough |  | Outside commitments  None  Some |  |
