# Supplementary material for: A review of the application and contribution of discrete choice experiments to inform human resources policy interventions
Source: Hum Resour Health. 2009 Jul 24;7:62. doi: 10.1186/1478-4491-7-62 (PMC2724490; doi:10.1186/1478-4491-7-62)
Supplement: Additional file 4 — Ranking of attributes according to their importance. Microsoft Word table in landscape format. [file 1478-4491-7-62-S4.doc]

### Additional file 4. Ranking of attributes according to their importance

| **Authors and year of publication** | **Ref.** | **Most**  **important**  **←** |  |  |  |  |  |  |  |  |  | **Least important**  **→** |
| --- | --- | --- | --- | --- | --- | --- | --- | --- | --- | --- | --- | --- |
| Gosden et al. 2000^a^ | [61] | Treating highly deprived patients (-) | Working with an extended team (+) | Opportunity to develop specialist interests (+) | Treating moderately deprived patients (-) | More daytime hours (-) | Number of out-of-hours work (-) | List size  (-) | Change in annual income (per £1 increase) (+) |  |  |  |
| Scott, 2001 ^b^ | [44] | More out of hours (average compared to none) (-) | More out of hours (large compared to average) (-) | Greater list size per GP (-) | Availability of guidelines (+) | Increase in annual income (+) | Daytime hours at work (-) | Time devoted to administration (-) | Special interests (+) |  |  |  |
| Ubach et al. 2003 | [63] | More on-call duties (-) | Possibility to do non-NHS work (+) | Better working relationships with the staff (+) | Shortage of staff (-) | Increased hours of work (-) | Increase in annual salary (+) |  |  |  |  |  |
| Wordsworth, 2004 | [62] | More out of hours (high intensity) (-) | More out of hours (medium intensity) (-) | Continuing professional development (+) | Extensive involvement in practice decisions (+) | Some involvement in practice decisions (-) | Consultation length (+) | Some outside commitments (+) | Change in hours of work/week (-) | Change in annual earnings (+) |  |  |
| Penn-Kekana et al, 2004 | [41] | Double salary(+) | Better facility management (+) | Fully equipped facility (+) | Salary increase of 15% (+) | Well staffed facility (+) | Developed social amenities (+) |  |  |  |  |  |
| Mangham & Hanson, 2007 | [43] | Pay increase from K40k to K50k (+) | Opportunity to upgrade (+) | Provision of basic housing (compared to none) (+) | Pay increase from K30k to K40k (+) | Job located in a city (vs. district towns) (+) | Decreased workload (+) | Better availability of resources (+) | Provision of superior housing (compared to basic) (+) |  |  |  |
| Hanson and Jack 2008  (doctors) | [64] | Being able to work in the private sector (+) | Pay increase (+) | Improved housing (+) | Job located in Addis (vs. other regional capitals) (+) | Better equipment and drugs in the facility (+) | Increased commitment in the public sector (-) |  |  |  |  |  |
| Hanson and Jack, 2008  (nurses) | [64] | Pay increase (+) | Job located in a city (vs. rural area) (+) | Better equipment and drugs in the facility (+) | Improved housing (+) | Better supervision (+) | Increased commitment in the public sector (-) |  |  |  |  |  |
| Kolstad 2008^c^ | [65] | Salary TSH650k (+) | Education opportunity after 2 years (+) | Salary TSH500k (+) | Education opportunity after 4 years (+) | Better infrastructure (+) | Sufficient equipment (+) | Education after 6 years (+) | Salary TSH350k (+) | Dar-es-Salaam (-) | District headquarters (+) | Housing improved (+) |

Note: This table is constructed with the absolute value of the regression coefficients obtained in the studies. The results indicate the direction of the effect on the likelihood to accept a job is given between parentheses.

No results are reported for Chomitz et al. (1998) because the authors do not report an analysis for their entire sample. They performed four different analyses for four subgroups, which makes it impossible to compare the results with one another.

a Increased out-of-hour work, change in annual income, absence of financial responsibility and change in list size were not statistically significant.

b Possibility to allocate some time for special interest or the time spent on administrative tasks were not significant attributes.

c Reduced workload and being based in regional headquarters were not significant attributes.
